# A Web-Based Non-Intrusive Ambient System to Measure and Classify Activities of Daily Living

**DOI:** 10.2196/jmir.3465

**Published:** 2014-07-21

**Authors:** Reto A Stucki, Prabitha Urwyler, Luca Rampa, René Müri, Urs P Mosimann, Tobias Nef

**Affiliations:** ^1^Gerontechnology and Rehabilitation GroupUniversity of BernBernSwitzerland; ^2^University Hospital of Old Age PsychiatryUniversity of BernBernSwitzerland; ^3^Perception and Eye Movement LaboratoryDepartments of Neurology and Clinical ResearchUniversity Hospital InselspitalBernSwitzerland; ^4^Gerontechnology and Rehabilitation GroupARTORG Center for Biomedical Engineering ResearchBernSwitzerland; ^5^ARTORG Center for Biomedical Engineering ResearchUniversity of BernBernSwitzerland

**Keywords:** ADL classifier, forward chaining inference engine, rule-based, wireless sensor system, dementia, Alzheimer, behavior pattern, activity monitoring, assistive technology, smart homes

## Abstract

**Background:**

The number of older adults in the global population is increasing. This demographic shift leads to an increasing prevalence of age-associated disorders, such as Alzheimer’s disease and other types of dementia. With the progression of the disease, the risk for institutional care increases, which contrasts with the desire of most patients to stay in their home environment. Despite doctors’ and caregivers’ awareness of the patient’s cognitive status, they are often uncertain about its consequences on activities of daily living (ADL). To provide effective care, they need to know how patients cope with ADL, in particular, the estimation of risks associated with the cognitive decline. The occurrence, performance, and duration of different ADL are important indicators of functional ability. The patient’s ability to cope with these activities is traditionally assessed with questionnaires, which has disadvantages (eg, lack of reliability and sensitivity). Several groups have proposed sensor-based systems to recognize and quantify these activities in the patient’s home. Combined with Web technology, these systems can inform caregivers about their patients in real-time (eg, via smartphone).

**Objective:**

We hypothesize that a non-intrusive system, which does not use body-mounted sensors, video-based imaging, and microphone recordings would be better suited for use in dementia patients. Since it does not require patient’s attention and compliance, such a system might be well accepted by patients. We present a passive, Web-based, non-intrusive, assistive technology system that recognizes and classifies ADL.

**Methods:**

The components of this novel assistive technology system were wireless sensors distributed in every room of the participant’s home and a central computer unit (CCU). The environmental data were acquired for 20 days (per participant) and then stored and processed on the CCU. In consultation with medical experts, eight ADL were classified.

**Results:**

In this study, 10 healthy participants (6 women, 4 men; mean age 48.8 years; SD 20.0 years; age range 28-79 years) were included. For explorative purposes, one female Alzheimer patient (Montreal Cognitive Assessment score=23, Timed Up and Go=19.8 seconds, Trail Making Test A=84.3 seconds, Trail Making Test B=146 seconds) was measured in parallel with the healthy subjects. In total, 1317 ADL were performed by the participants, 1211 ADL were classified correctly, and 106 ADL were missed. This led to an overall sensitivity of 91.27% and a specificity of 92.52%. Each subject performed an average of 134.8 ADL (SD 75).

**Conclusions:**

The non-intrusive wireless sensor system can acquire environmental data essential for the classification of activities of daily living. By analyzing retrieved data, it is possible to distinguish and assign data patterns to subjects' specific activities and to identify eight different activities in daily living. The Web-based technology allows the system to improve care and provides valuable information about the patient in real-time.

## Introduction

The number of older adults in the global population is increasing both in absolute and relative terms due to increased life expectancy [1]. This demographic shift has led to an increasing prevalence of age-associated disorders, such as Alzheimer’s disease and other types of dementia. According to the World Health Organization, today there are 35 million patients worldwide suffering from dementia. By the year 2030, the number of patients is expected to double to 66 million worldwide [2]. Alzheimer’s disease is a neurodegenerative disease, and with its progression, the ability to cope with activities of daily living decreases, leading to reduced autonomy and increased need for care [3]. With the progression of the disease, the risk of institutional care increases, which contrasts with the desire of most patients who would like to live autonomously in their familiar home environment as long as possible [4]. Despite awareness of the patient’s cognitive status by doctors and caregivers, they are often uncertain about its consequences on activities of daily living (ADL). To provide effective care, they need to know how good patients cope with ADL, in particular when it comes to the estimation of risks associated with the cognitive decline. It is not yet clear how cognitive decline influences ADL. In this regard, the occurrence, performance, and duration of different ADL are important indicators of functional ability [3].

The patient’s ability to cope with ADL is traditionally assessed with questionnaires (eg, Stanford Health Assessment Questionnaire [5] or the Barthel ADL (activities of daily living) Index [6]), and it is important information for professional caregivers in order to optimize medication and personalize care [3]. However, using questionnaires to assess behavioral data in patients with cognitive impairments has disadvantages (eg, lack of reliability and sensitivity), and several groups have proposed sensor-based systems to recognize and quantify ADL [4,7-9] in the patient’s home. Besides storing the occurrence, duration, and location of ADL, when combined with Web technology, these systems can also inform caregivers in real-time (eg, via smartphone) about the current ADL of their patients [7-10]. Moreover, current and future clinical trials of new drug interventions, in Alzheimer’s disease will need to prove their effects on ADL function, and sensitive and reliable measurements will be of great importance. Also, it has been suggested that such systems could facilitate and support aging-in-place and improve medical care [9,10].

One possible technical solution for recognizing ADL is using comprehensive sensor networks to measure patient’s activities. In so-called smart homes, several sensors, for example, accelerometers, microphone arrays, pressure sensitive mats, gas sensors, and cameras are installed in the proximity of older patients to determine specific activities and to monitor their ability to cope with ADL [7,8]. Commercial smart-home monitoring systems for dementia patients are already available on the market. For example, ALARM-NET and CareWatch have been developed to recognize ADL and to improve dementia care provided by both formal and informal caregivers [9,10]. Other technical systems make use of detachable sensor arrays, for example, wearable accelerometers, infrared sensors and pressure sensors in furniture, wireless heart rate monitors, or radio frequency technology. Such sensor networks are used for automated ADL classification in dementia patients to maintain their autonomy, for recognizing emergencies, and monitoring the progression of their disease [11-16].

However, state-of-the-art technology systems have some limitations. Smart homes, for example, are often very comprehensive and require significant installation efforts, which makes these systems costly and challenging to install in older houses [17]. Also, most smart home systems use video-based technology or microphone recordings, which might conflict with the patient’s privacy [18]. Other approaches require that the patient wear body-mounted sensors or interact with a system for data acquisition [11,12,15]. Using these systems requires patient compliance, which is not always assured when patients with cognitive impairments are overloaded by technology [19,20]. Furthermore, others have reported that too-intrusive systems are not well accepted by patients or caregivers [21,22].

We hypothesize that a non-intrusive system, which does not use body-mounted sensors, avoids video-based imaging and microphone recordings, and does not require any interaction or patient compliance would be better suited for use in dementia patients. If small, easy to install, and not in need of patient’s attention and compliance, such a system might be well accepted by patients [23]. Furthermore, such a system, due to its low cost, robustness, and easy-to-use approach, can be of interest for other researchers from different fields. ADL can be a relevant outcome measurement for future drug intervention studies.

In this paper, we present a novel passive, Web-based, non-intrusive, assistive technology system that recognizes and classifies ADL. Using the Web, the system can provide information on current patient behavior that can help to quantify the patient’s cognitive impairment, estimate the patient’s self-dependency, and facilitate formal and informal care of Alzheimer’s disease patients living alone. We present here the results of the new Web-based, non-intrusive wireless sensor system and an ADL classifier. We hope that the outcome of our study, together with further investigation and research, will justify further interventional studies.

## Methods

### Overview

The components of this novel assistive technology system are (1) a number of sensors that are distributed in every room and (2) a central computer unit (CCU). The collected data are stored and processed by an ADL classifier based on a rule-based forward chaining inference engine. Eight ADL, such as sleeping, grooming, toileting, getting ready for bed, cooking, eating, watching TV, and seated activity were allotted for the ADL classifier to determine. The ADL were chosen in consultation with medical experts. Table 1 shows the eight ADL in detail, along with a definition for each.

**Table 1:** The eight ADL in detail, with definitions.

ADL	Definition
Sleeping	Includes: Night rest, taking a nap (either in bed or on the couch). Excludes: Lying down (not sleeping) for recovery.
Grooming	Includes: Personal hygiene: Showering, toileting, shaving, brushing teeth, and styling as one activity. Excludes: Simple toileting and hand washing.
Toileting	Includes: Simple toileting with washing hands. Excludes: Other or additional personal hygiene.
Getting ready for bed	Includes: Personal hygiene before bedtime. Excludes: Pre-bedtime rituals.
Cooking	Includes: Preparing food in the kitchen. Excludes: Cutting pizza from delivery service, making popcorn, etc, making tea or coffee.
Eating	Includes: Having a meal (also delivered food). Excludes: Snacking (eg, while watching TV), having just a cup of coffee or a glass of water.
Watching TV	Includes: Watching TV with main focus on the TV. Excludes: Other activities while the TV is just on.
Seated activity	Includes: Sitting at a table or in an easy chair while reading, solving a puzzle, doing crosswords, embroidering, doing crafts, or listening to the radio. Excluding: Taking a nap.

### Participants

Healthy participants were recruited by advertisements in local newspapers. They were assessed with a standardized paper-pencil test battery, which included the Montreal Cognitive Assessment (MoCA) [24,25], the Trail Making Test A and B (TMT-A, TMT-B) [26], and the Timed Up and Go test [27,28]. Exclusion criteria for the study were cognitive impairment (MoCA score < 26), or significant motor impairment (Timed Up and Go > 12 seconds). Participants sharing the house with others and not living alone were also excluded.

In total, 10 healthy mobile adults (6 women, 4 men; age 28-79 years) were included in the study. The study was carried out in accordance with the Declaration of Helsinki and was approved by the local ethics board. Written informed consent was obtained from all participants prior to inclusion. No compensation for participation was provided.

### The Assistive Technology System

**Figure 1:**
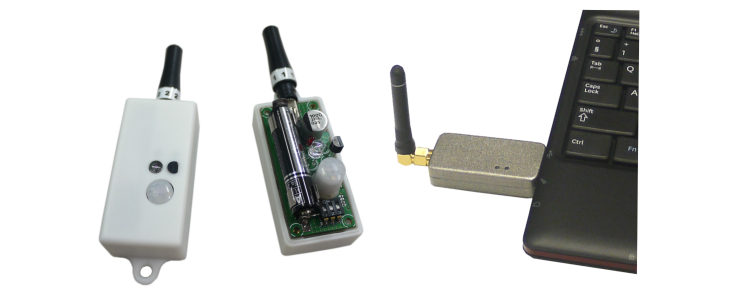
Wireless sensor box with housing (left) and an inside view of the same sensor box (middle) displaying PCB board with environmental sensors and primary cell; receiver unit with antenna (right) is connected to the central computer unit serving as a data server and Web link (between the two devices a bidirectional data transmission is established).

In each case, five sensors were mounted on a printed circuit board (PCB) capturing ambient values: temperature in °C (DS18B20, Dallas Inc), humidity in g/m^3^ (SHT21P, SENSIRION), luminescence in lx (AMS302, Panasonic Inc), motion (binary) (EKMB1101111, Panasonic Inc), and acceleration in m/s^2^ (ADXL345, Analog Device). Each PCB was assembled in polyvinyl chloride (PVC) housing (Figure 1) and was powered by a 2900 mA AAA primary cell.

In total, 50 sensors were assembled to 10 wireless sensor boxes (*l* x *w* x *h*=15mm x 30mm x 60mm, weight=80g), which digitalizes the analog environmental data and sends it to the CCU. A commercial available laptop, running customized Microsoft Windows 7, builds the CCU and served as data server with integrated Web link (Figure 1). The receiver unit (Figure 1), which is attached to the CCU, collected the data packages sent from all 10 sensor boxes. It comes with a digital received signal strength indication, an input sensitivity of 117 dBm, and a programmable transmitter output power up to 13 dBm. The CCU has the computing power to process the environmental data but also to store the data for further analysis.

The environmental data were acquired at a sampling rate of 0.2 Hz and assembled to data packages. Beside the five environmental values, each data package includes a handshake word composed of timestamp, date, node number, supply voltage, and a status word. The data packages were sent to the data server over an air link based on EZRadioPro-Technology (Silabs). Therefore, a low byte order marked bidirectional protocol placed on an 868 MHz carrier transmitted by frequency modulation was implemented. Even-parity error handling and frame collision detection was implemented.

### System Set-up

**Figure 2:**
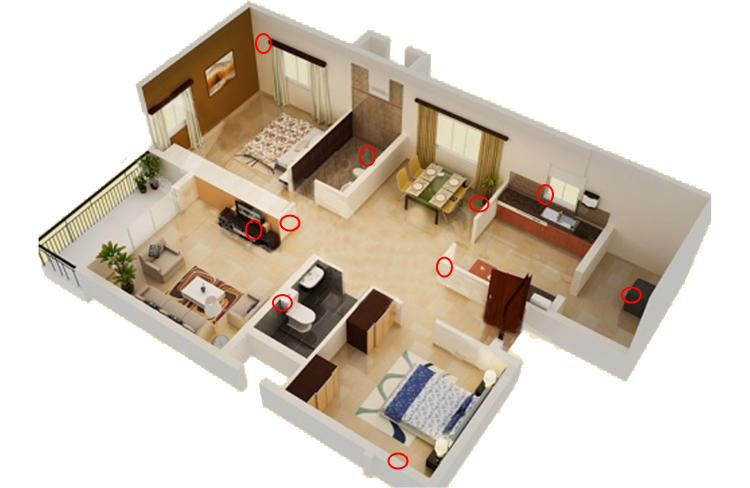
Example setup installed in a 2-bedroom apartment (sensor boxes indicated with red circles).

The assistive technology system was installed in the home of 10 healthy subjects. Figure 2 shows the floor plan of a sample 2-bedroom apartment. In each apartment, each compartment (room) was fitted with one sensor box, placed at a height of approximately 2 meters, facing towards the middle of the room. Additional sensor boxes were placed in the kitchen (on the fridge door) and in the bathroom (on the flush handle).

Installation duration for the entire system depends on the apartment layout and varied from 15-30 minutes. Once set up and initialized, the system continuously recorded the five ambient environmental values autonomously. Activities of the 10 subjects were recorded for 20 days each. The subjects were told to ignore the system as much as possible and act naturally. They were also asked to touch the sensor boxes only if absolutely necessary. The system was uninstalled after 20 days of continuous measuring.

### Subject Log Book

**Figure 3:**
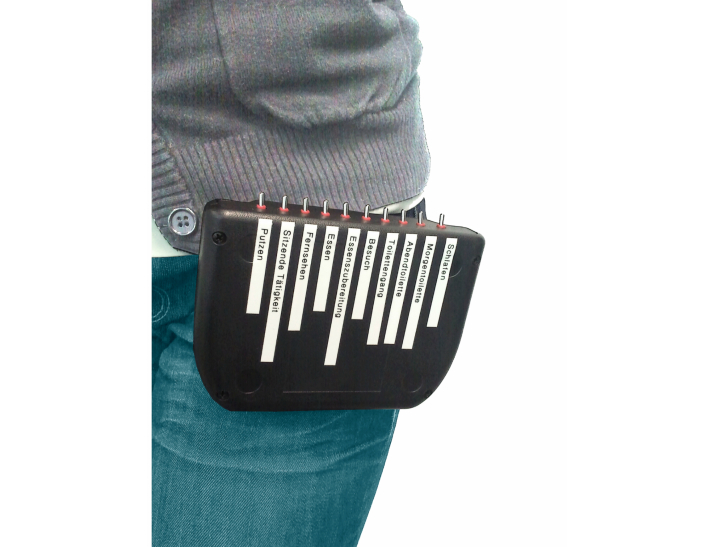
Wireless protocol device worn by participant (switches on top of the box are labeled with 8 different activities such as "watching TV" or "sleeping").

For evaluation purposes, a wireless protocol device, built in a housing with a wearable belt clip, was provided to the subjects (Figure 3). The protocol device was fitted with switches, each corresponding to a specific ADL. The ten switches provided on the protocol box were “Sleeping”, “Grooming”, “Toileting”, “Getting ready for bed”, “Cooking”, “Eating”, “Watching TV”, “Seated activity”, “ receiving visitors”, and “cleaning”. Of the ten switches, one activity (cleaning) and one state (receiving visitor) were not included in the analysis. All subjects were instructed to record their activities throughout the day for the duration of the 20 days measurement. For this purpose, they were asked to flick the corresponding switch, labeled on the protocol device case, during the performance (exact duration) of the given ADL. The protocol device was intended to be worn during the day. Only if the subjects were disturbed by wearing the device (eg, during nighttime or when grooming) were they allowed to take it off.

In addition, participants were provided with a paper-pencil log book, to record every wrongly stated, forgotten, or missed ADL.

### Data Processing

**Figure 4:**
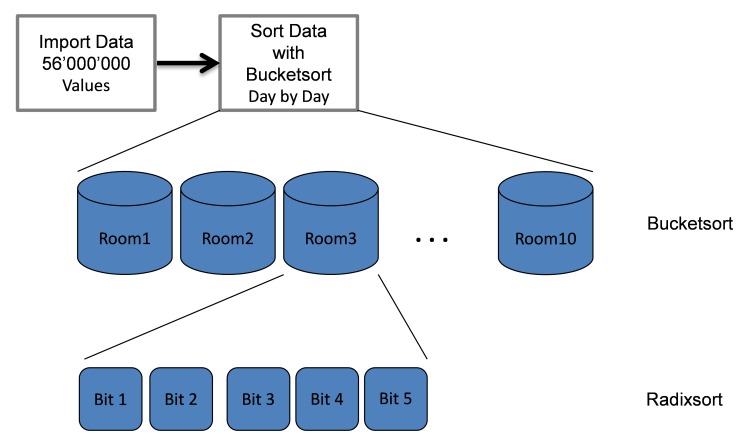
Combined sort algorithm consists of a Bucketsort and a Radixsort algorithm applied to the data.

First, two different sorting methods were used sequentially to increase the sorting efficiency of the acquired data (Figure 4). A Bucketsort algorithm [29] was first applied to the data, which were labeled with a compartment identifier by the system during acquisition. Each data value was allotted to the compartment (room) where the value was captured. Metaphorically, each compartment was represented by one bucket. The data in each bucket were then sorted with a Radixsort [30] algorithm. The five bits considered by the Radixsort are related to the intra-day-timeline. This sorting of data is needed to line up the measured values chronologically.

In summary, the data are bijectively matched to their origin compartment by the Bucketsort, and then lined up in a chronological order by Radixsort. A mathematical description regarding the efficiency of the two sort algorithms is presented in Multimedia Appendix 1, and the open source code is provided in Multimedia Appendix 2.

**Figure 5:**
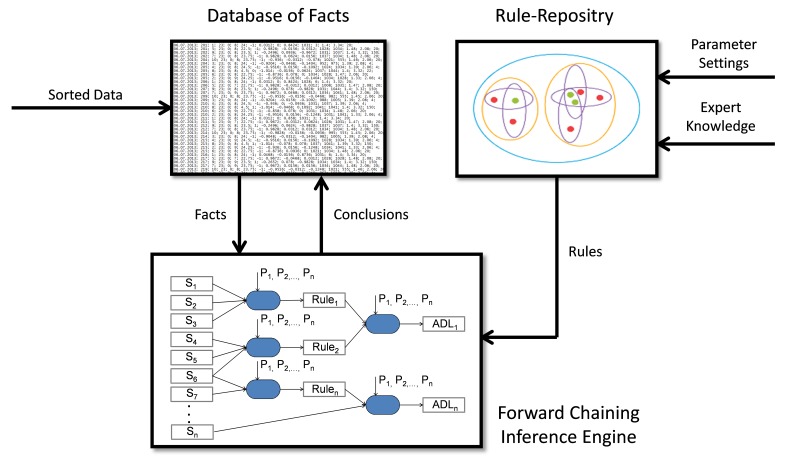
Block diagram of the ADL classifier algorithm based on a forward chaining inference engine.

In the second step, the data were classified using an ADL classifier (Figure 5). The classification of the ADL is based on the assumption that each subject follows a daily routine, where specific patterns with nearly the same duration and course occur throughout each day [31]. Considering the fact that numerous data values are accumulating during the measurements, the classifier was implemented as a rule-based inference engine. The concept is ideal to handle numerous data, as it is widely used in the field of very-large-scale integration [32]. The theoretical concept was adapted to match the needs of the ADL classifier. It consists of (1) a database, (2) a rule-repository, and (3) a forward chaining inference engine. The database (1) holds all the data sorted upfront by the Bucketsort and Radixsort algorithms, but also all the classified ADL so far (historical data). The rule-repository (2) provides the forward chaining inference engine with a set of parameterized behavioral knowledge (the parameterization was done in cooperation with our medical experts.) A parser translates the parameterized behavioral knowledge into a look-up table disposable in the random access memory. The (3) forward chaining inference engine charges all available facts according to the given rules. The rules were defined manually and were the same for all subjects. The daily routine itself (ie, the time of the activity) was not considered within the rules; rather, the rules were applied to the daily routine resulting from the specific behavior pattern throughout the day. Nevertheless, the forward chaining inference engine needs some conflict resolution strategy to decide which information is the most important to process first and in which order the rest of the information has to be taken into account. Going the other way, the forward chaining inference engine checks which condition information must be fulfilled to state the given information as one specific ADL.

The used method is to obey the rules that the medical experts defined first. The forward chaining inference engine, on the other hand, works with three basic elements: (1) ambient value matrices, (2) rules, and (3) behavioral parameters. For each potential ADL, an ambient value matrix is composed of the sorted raw data. Each ambient value matrix is then compared to a set of rules defined upfront for each of the eight ADL. The rules are built under the premise of the behavioral parameter. If the ambient value matrix fits the set of rules, the activity is considered as the corresponding ADL.

For example, if one looks at “toileting”, the matrix consists of the data from the five sensors in the bathroom and from the flush handle. Hence, the ambient value matrix shows a specific pattern associated with toileting. First, the forward chaining inference engine verifies the activity’s duration for toileting. Further, the forward chaining inference engine verifies if the light conditions in the bathroom change, if the subjects sit on the toilet, if the subjects flushes, and if the humidity and the temperature do not change significantly. Having these prerequisite (rules) fulfilled, the activity is stated as “toileting”. The mathematical formulas and the source code of the forward chaining inference engine are provided in Multimedia Appendix 3.

Depending on the complexity of the ADL to be classified, n-steps of iteration can be done. By processing the daily routine with the developed classifier algorithm, the system allots specific patterns to one of the 8 selected ADL. The output of the classifier (the conclusion) is the ADL a subject performed at a given time throughout the day. This information is stored in the database.

The calculation and algorithms were implemented in Matlab R2007b (MathWorks, Inc).

### Data Analysis

**Figure 6:**
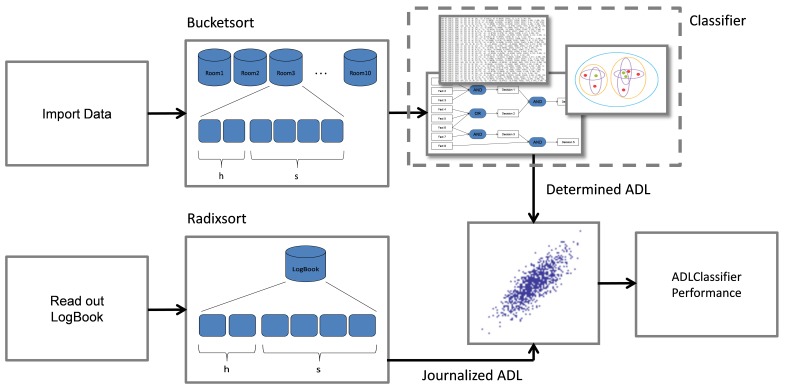
Correlation of the ADL classifier output with the ADL protocols.

The subject log book was updated by superimposing the data of the wireless protocol device with the paper-pencil log book. The systems’ performance (sensitivity and specificity) was then calculated by correlating the output of the ADL classifier with the ADL protocols (Figure 6).

### Data Collection With an Alzheimer Patient

In addition to the 10 healthy subjects, one dementia patient was measured for explorative purpose. This was done to understand the acceptance of the system in the target group of dementia patients and also to estimate the further clinical implications. The measurement was performed similarly in all respects to the measurement in the healthy subjects. The patient was an 84-year-old female. She was receiving home care twice a week for 1 hour, and she was on her own for the rest of the time.

## Results

### Demographics

Table 2 shows the demographics of 10 healthy participants (6 women, 4 men; mean age 48.8 years; SD 20.0 years; age range 28-79 years) included in the study. The test performance in MoCA (score range 27-30), TMT-A (time range 15.3-81.4 seconds), TMT-B (time range 21.1-122.6 seconds), and Timed Up and Go test (time range 6.9-10.3 seconds), of all the participants were in a normal, non-pathological range.

**Table 2:** Demographics of 10 healthy participants included in the study.

Characteristics		Value
**Gender, n**		
	Male	4
	Female	6
**Age, years**		
	Minimum	28.0
	Maximum	79.0
	Mean	48.8
	SD	20.0
**MoCA Score (Maximum = 30)**		
	Mean	29.1
	SD	1.1
**Timed Up & Go**		
	Mean	8.2
	SD	1.3
**Trail Making Test (TMT)**		
	Mean TMT A	39.1
	SD TMT A	20.0
	Mean TMT B	62.6
	SD TMT B	32.3
**Measured time, days**		
	Mean	20.0
	SD	0.0

### Data Analysis and Activities of Daily Living Classifier Performance

In total, 33,939,441 data packets (543,031,056 environmental values) were captured correctly, and 160,269 data packets (0.47%) were lost due to transmission error. Thus, an overall reliability of 99.53% was achieved.

In total, 1317 ADL were performed by the participants, 1211 ADL were classified correctly, and 106 ADL were missed. This leads to an overall sensitivity of 91.27% and a specificity of 92.52% (Table 3). Each subject performed 134.8 ADL on average (SD 75).

**Table 3:** Results of the ADL classifier.

ADL	N	Classified correctly, n	Missed, n	Sensitivity, %	Specificity, %
Sleeping	173	161	12	93.64	85.77
Grooming	135	127	8	94.07	96.98
Toileting	307	291	16	94.79	91.54
Getting ready for bed	105	97	8	92.38	94.48
Cooking	70	59	11	84.29	90.92
Eating	90	78	12	87.78	94.83
Watching TV	322	300	22	93.17	90.63
Seated activity	171	151	20	90.06	94.98
Total	1317	1211	106	91.27	92.52

### Behavior Pattern of an Alzheimer Patient Versus a Healthy Subject

**Figure 7:**
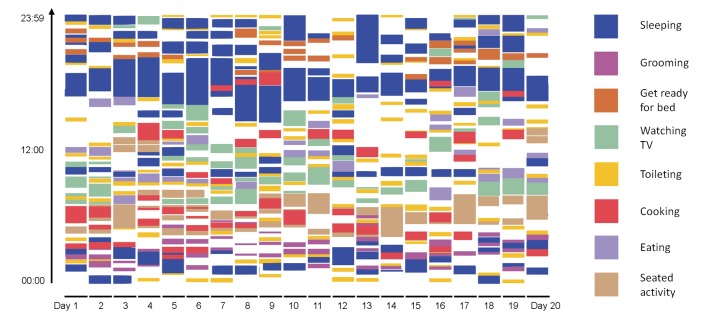
Activity map of 84-year-old female Alzheimer patient.

Figure 7 shows the activity pattern for the 84-year-old female Alzheimer patient (MoCA score=23, Timed Up and Go=19.8 seconds, Trail Marking Test A=84.3 seconds, Trail Marking Test B=146 seconds).

**Figure 8:**
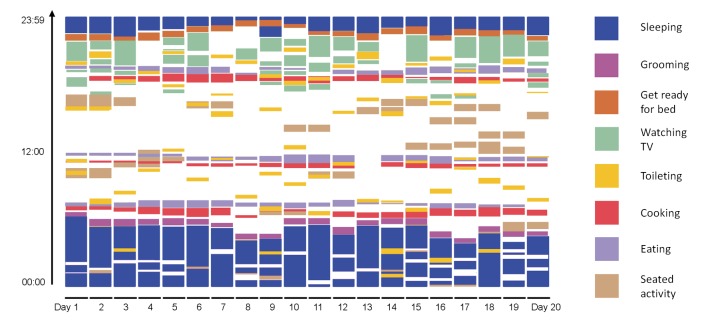
Activity map of healthy 79-year-old female.

The activity pattern of a healthy female (age=79, MoCA score=29, Timed Up and Go=12.7 seconds, Trail Marking Test A=62.2 seconds, Trail Marking Test B=95.1 seconds) is shown in Figure 8.

Figures 7 and 8 show the duration and points in time of the performed ADL of the Alzheimer patient versus the healthy subject.

## Discussion

### Principal Findings

The feasibility and reliability of the newly developed non-intrusive sensor system (hardware and the operating software) could be proven in the field. The results show that daily living evokes variation in ambient values that can be captured by the sensor system, leading to ADL specific data patterns. Hence, it is possible to assign sensor data patterns to specific activities, whereby the most relevant sensor in the detection of ADL is the movement sensor. By analyzing the retrieved data, it became possible to identify eight ADL.

Other researchers have conducted similar studies. Bang et al [12] used a set of pressure sensors, passive infrared sensors, and a worn accelerometer to determine ADL. Depending on the specific ADL, they achieved an initial accuracy of 93.27% to 96.47%. Fleury et al [16] used video cameras and wearable kinetic sensors, door contacts, and microphones to acquire data. The data were processed by a support vector machine to determine specific ADL. They achieved a sensitivity of 97.80%. Both results are slightly better than what we found—at the cost of more intrusive sensor systems.

The ADL classification performance of the system relied on the protocol compliance of the subjects during the measurement. We asked the subjects to record their ADL very carefully with the electronic protocol device and provided them furthermore with paper-based log books to note any errors or wrong manipulations. Overall, it is possible that subjects forgot to record an activity or recorded a wrong activity, which negatively affects the classification performance numbers. Hence, the reported performance of the ADL classifier expressed by sensitivity and specificity numbers actually represents worst-case scenario.

A limitation of our system is that the sensors need a clear view of the entire room and must not be covered. This is can be solved by proper placement of the sensors in the patient’s home.

As expected, the classifier showed better performance for some ADL (eg, grooming) than it did for others (eg, sleeping). This is due to the fact that some ADL are not exclusively room related and evoke only slightly different ambient values. Such data pattern differs only poorly among one another, which has also been found by other researchers [12,16,33].

The system uses wireless technology with small, discrete shaped sensor boxes. Hence, no hardware or tools are needed for the installation and the set-up can be done in less than 30 minutes. All parts were built under consequent low-power conditions, which keeps the system in service for 6 months straight. During this period, no maintenance is needed. Once the system is set up, the functioning of each sensor box can be monitored at any time. This leads to a high degree of transparency regarding the transmitted, lost, and compromised data (reliability of 99.53%). Furthermore, data can be bijectively matched to its origin to take statistical advantage of paired data, which ensures valid data throughout the measurement. Moreover, by foregoing the use of cameras and microphones, the privacy of the subjects can be guaranteed at any time of the experiment. Furthermore, due to its Web link, the system can provide discrete information about the patient’s ADL performances in real-time, but also historical and statistical information about the progression of the disease.

When it comes to measurement in dementia patients, we proved feasibility in one female Alzheimer patient. The acceptance of the system was good, and no significant incident or failure occurred during the measured period. The patient was not disturbed by the system.

The activity map of the patient shows significant difference to the activity map of the healthy subject. While in the healthy subject, a daily structure is observable, much less structure can be found in the Alzheimer patient. Comprehensive analysis of further datasets of dementia patients can provide valuable clinical information, especially by comparing it to datasets of healthy patients.

These preliminary results obtained from one Alzheimer patient are promising in anticipation of further research and the clinical implication of the system.

### Conclusions

The non-intrusive wireless sensor system can be used to acquire environmental data essential for the classification of ADL. By analyzing the retrieved data, it is possible to distinguish and assign data patterns to subjects’ specific activities and to identify eight different ADL. Thanks to the Web-based technology, the system has a high potential to improve care and provides valuable information about the patient in real-time.

Based on the results of this study, there are plans to install the system in the home of Alzheimer patients, suffering from moderate to severe dementia. The data of these patients will be compared to the data of the healthy subjects to study and analyze the different behavior patterns.

## Multimedia Appendix 1

Bucketsort and Radixsort formulas.

## Multimedia Appendix 2

Source code.

## Multimedia Appendix 3

Forward Chaining Inference Engine formulas.

## Abbreviations

ADL: activities of daily living
CCU: central computer unit
MoCA: Montreal Cognitive Assessment
PCB: printed circuit board
PVC: polyvinyl chloride
TMT A & B: Trail Making Test A and B
