# Supplementary material for: A Web-Based Non-Intrusive Ambient System to Measure and Classify Activities of Daily Living
Source: J Med Internet Res. 2014 Jul 21;16(7):e175. doi: 10.2196/jmir.3465 (PMC4129128; doi:10.2196/jmir.3465)
Supplement: Supplementary file 1 [file jmir_v16i7e175_app1.pdf]

Formulas of Bucketsort and Radixsort

EfficiencyBS= On+i=0n-1 Oni2

Due to the fact that all ambient values were recorded in a numerical format, a Radixsort algorithm was applied to sort the values in each “Bucket”. Its efficiency *O* is

EfficiencyRS = O(m*n) | m=5

For the whole sortation, an overall efficiency *O* of

Efficiency = On+i=0n-1O5n

can be achieved.
