# Supplementary material for: A Web-Based Non-Intrusive Ambient System to Measure and Classify Activities of Daily Living
Source: J Med Internet Res. 2014 Jul 21;16(7):e175. doi: 10.2196/jmir.3465 (PMC4129128; doi:10.2196/jmir.3465)
Supplement: Supplementary file 3 [file jmir_v16i7e175_app3.pdf]

Formulas of the Forward Chaining Inference Engine

Ambient values matrices = S1tend-tstart,S2tend-tstart,…,Sitend-tstart

Behavioural parameter = P1,P2,…,Pj

Rules = R1,R2,…,Rk

Activities of daily living = ADL1,ADL2,…,ADL8

R1=(S1tend-t0>P1) □ (S2tend-t0=P2) □ … □ (Sitend-t0≥Pj)
R2=(S3tend-t0≤P3) □ (S1tend-t0>P4) □ … □ (Sitend-t0=Pj)
.
.
.
Rk=(S2tend-t0=P4) □ (S4tend-t0≤P2) □ … □ (Sitend-t0≠Pj)
